# Clinicopathologic characteristics and prognostic factors of ovarian fibrosarcoma: the results of a multi-center retrospective study

**DOI:** 10.1186/1471-2407-10-585

**Published:** 2010-10-27

**Authors:** Long Huang, Ling-Min Liao, Hui-Yun Wang, Min Zheng

**Affiliations:** 1 Department of Gynecology, Cancer Center, Sun Yat-sen University, Guangzhou 510060, PR China; 2 State Key Laboratory of Oncology in Southern China, 651 Dongfeng Road East, Guangzhou 510060, PR China; 3 Guangdong Key Laboratory of Medical Molecular Imaging, Shantou 515041, PR China; 4 Department of Radiology, First Affiliated Hospital, Medical College of Shantou University, PR China

## Abstract

**Background:**

Ovarian fibrosarcomas are very rare tumors, and therefore, few case studies have evaluated the prognostic factors of this disease. To our knowledge, this study represents the largest study to evaluate the clinical and pathologic factors associated with ovarian fibrosarcoma patients.

**Methods:**

Thirty-one cases of ovarian fibrosarcoma were retrospectively reviewed, which included medical records for eight patients, and 23 published case reports from 1995 through 2009. Patient treatment regimens included total hysterectomy with bilateral adnexectomy and an omentectomy (BAO) (n = 9), oophorectomy (OR) (n = 8), chemotherapy (CT) (n = 1), BAO followed by chemotherapy (BAO+CT) (n = 11), BAO followed by radiotherapy (BAO+RT) (n = 1), and oophorectomy followed by radiotherapy (OR + RT) (n = 1).

**Results:**

The patients of this cohort were staged according to the guidelines of the Federation of Gynecology and Obstetrics (FIGO), with 15, 6, 9, and 1 stage I-IV cases identified, respectively. Mitotic count values were also evaluated from 10 high-power fields (HPFs), and 3 cases had an average mitotic count < 4, 18 cases were between 4 and 10, and 10 cases had an average mitotic count value ≥ 10. The Ki-67 (MIB-1) proliferation index values were grouped according to values that as follows: < 10% (n = 5), between 10% and 50% (n = 9), and ≥ 50% (n = 5). Positive expression of vimentin (100%, 22/22) and negative expression of CD117 (0%, 5/5) were also detected. Moreover, expression of smooth muscle actin (2/18), desmin (1/13), epithelial membrane antigen (0/11), S-100 (1/19), CD99 (0/6), CD34 (1/5), α-inhibin (7/15), estrogen receptor (1/6), and progesterone receptor (1/6) were reported for subsets of the cases examined. After a median follow-up period of 14 months (range, 2-120), the 2-year overall survival rates (OS) and disease-free survival (DFS) rates for all patients were 55.9% and 45.4%, respectively. Cox proportional hazard regression analysis of survival showed that FIGO stage (*P *= 0.007) and treatment (*P *= 0.008) were predictive of poor prognosis. Furthermore, patients with stage I tumors that received BAO+CT were associated with a better prognosis.

**Conclusions:**

Mitotic activity, and cells positive for Ki-67 were identified as important factors in the diagnosis of ovarian fibrosarcoma. Furthermore, FIGO stage and treatment modalities have the potential to be prognostic factors of survival, with BAO followed by adjuvant chemotherapy associated with an improved treatment outcome.

## Background

Ovarian brosarcomas primarily originate in the ovaries [1], and are very rare tumors. Correspondingly, very few cases of ovarian fibrosarcoma have been reported. As a result, ovarian fibrosarcomas can be difficult to distinguish clinically and histologically from cellular fibromas [1-3]. The malignant potential of ovarian fibrosarcomas are usually assessed based on observed growth patterns, cellular atypia, and mitotic counts [2]. However, due to the low incidence of this disease, it is difficult to enroll a sufficient number of patients to evaluate the prognostic factors associated with survival. Furthermore, since an optimal treatment strategy for this aggressive tumor has not yet been identified, it is not only difficult to treat patients with ovarian fibrosarcoma, but most patients with this disease do not survive more than 2 years due to early metastasis via the bloodstream and tumor recurrence [4]. As a result, the prognosis for patients with fibrosarcoma is very poor [1,2,5], and there are few reports regarding the long-term survival of patients with ovarian fibrosarcoma.

Therefore, in this study, a retrospective multi-center clinical trial was established to determine whether clinical or pathologic prognostic factors can be identified for patients with ovarian fibrosarcoma.

## Methods

A total of 31 patients diagnosed with ovarian fibrosarcoma were identified from various sources. Three of these cases were treated at the First Affiliated Hospital, Medical College of Shantou University (Guang Dong, China) and 5 cases were treated at the Cancer Center, Sun Yat-sen University (Guang Dong, China). In addition, 5 case reports published in a Chinese journal [6-10], and 18 cases reported in the PubMed database between January 1995 and December 2009 were retrospectively examined. Institutional review board approval was obtained from each of the participating centers.

Histopathologic diagnosis was based on morphologic criteria and immunohistochemical staining. Patients from the Medical College of Shantou University (n = 3) and the Cancer Center, Sun Yat-sen University (n = 5) were staged according to the FIGO clinical staging system for ovarian cancer, and included information from physical examinations, chest X-rays, intravenous (i.v.) pyelography, cystoscopy, sigmoidoscopy, abdomino-pelvic computed tomography (CT) scans, or magnetic resonance imaging (MRI). When suspicious findings were identified on chest X-rays and/or during physical examination, a CT scan of the chest and/or brain was performed.

Clinical and pathological variables analyzed included patient age, tumor size, FIGO stage, symptoms, mitotic events, immunohistochemical results, and treatment modalities. The primary end point was any cancer-related death. End points were calculated from the date of surgery for the cases from the Medical College of Shantou University and the Cancer Center, Sun Yat-sen University, while a presumed date of surgery was estimated for the additional 23 cases reviewed from the literature.

Overall survival (OS) and disease-free survival (DFS) were evaluated using the Kaplan-Meier method and log-rank tests. The Cox proportional hazards model was used to estimate the independent factors prognostic for OS and DFS. All analyses were carried out using SPSS software (version 13.0, SPSS Inc., Chicago, IL), and a significance level of 0.05 was used. All end points were updated in March 2010.

## Results

### Clinical Features

**Figure 1:**
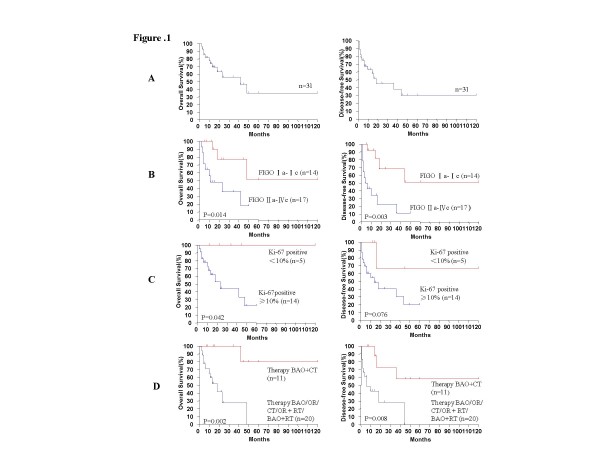
**Kaplan-meier survival curves for the clinicopathologic factors of patients with ovarian fibrosarcoma**. **(A) **Survival curves for the OS and DFS rates associated with 31 patients diagnosed with ovarian fibrosarcoma as indicated. **(B) **Survival curves for OS and DFS rates in relation to various FIGO stages of ovarian fibrosarcoma as indicated. **(C) **Survival curves for OS and DFS rates in relation to the percentage of Ki-67-positive cells detected (< 10% shown in red and ≥ 10% shown in blue). **(D) **Survival curves of OS and DFS rates associated with BAO/OR/CT/OR + RT/BAO+RT (shown in blue) vs. BAO + CT alone (shown in red).

In this cohort (n = 31), patient age ranged from 20 - 73 years, with a median age of 49 years (Table 1). For 6/25 (24%) patients, the ovarian fibrosarcomas were detected during a routine gynecological examination. Alternatively, 19/25 (76%) patients presented with abdominal pain, vaginal bleeding, or notable pelvic swelling at a hospital. For 6/31 cases that were reviewed from published case reports, the details of presentation were not provided [2]. FIGO staging of the tumors of this cohort included 15 (49%) stage I cases, 6 (19%) stage II cases, 9 (29%) stage III cases, and 1 (3%) stage IV case. Correspondingly, treatment for this cohort varied. Nine patients were treated with a total hysterectomy with bilateral adnexectomy and an omentectomy (BAO), 8 patients underwent an oophorectomy (OR), 1 patient received chemotherapy (CT), 11 patients received BAO followed by a chemotherapy (BAO+CT), and 2 patients underwent BAO followed by treatment with radiotherapy (BAO+RT). After a median follow-up period of 14 months (range, 2-120), the 2-year OS rates and 2-year DFS rates for this cohort were 55.9% and 45.4%, respectively (Figure 1A).

**Table 1:** The clinical and pathologic features of the ovarian fibrosarcoma cases examined

Reference	Case No.	Age	Symptom	FIGO stage	Largest diameter (cm)	Mitoses/10 HPFs	CD10	Ki-67 (MIB-1) positive	Vimentin	CD117	SMA	Des	EMA	S-100	CD99	CD34	α-inhibin	ER	PR	CA125 U/ml	Therapy	Recurrence			Follow-up (months)
																							
																						Interval (months)	Site	Treatment	
China Case 1[6]	1	43	AP	III c	10	≥ 4	(-)	50%-75%	(+)	(-)	(-)	/	/	(-)	(-)	/	(-)	(-)	(-)	/	OR	1	Pelvis	BAO	Died (4)
China Case 2 [7]	2	39	AP	I a	11	5~7	(-)	85%	(+)	/	(-)	(-)	(-)	(-)	(-)	/	(-)	(-)	(-)	/	BAO	NP	NP	NP	TF (14)
China Case 3	3	53	NP	I a	10	3~7	/	/	(+)	/	(-)	(-)	(-)	(-)	(-)	/	(-)	(-)	(-)	68.45	BAO	NP	NP	NP	TF (12)
China Case 4	4	20	NP	II a	7	3~5	/	15%	(+)	(-)	(-)	(-)	(-)	(-)	(-)	/	(-)	/	/	/	BAO	6	Pelvis	CT	Died (10)
China Case 5[8]	5	56	NP	III c	16	13	(-)	25%	(+)	(-)	(-)	(-)	(-)	(-)	(-)	(-)	/	/	/	< 35	BAO+(IA+DTIC)	NP	NP	NP	TF (8)
China Case 6[9]	6	43	NP	I a	5.7	16	(-)	> 10%	(+)	/	(-)	/	/	(-)	/	(-)	(+)	/	/	< 35	BAO+PVB	NP	NP	NP	TF (7)
China Case 7 [10]	7	21	AP	III b	15	≥ 4	/	> 10%	(+)	/	/	/	/	/	/	/	/	/	/	/	OR	3	Pelvis	NP	Died (6)
China Case 8	8	66	AP	II b	11	8	/	15%	(+)	/	(-)	(-)	(-)	(-)	/	/	(-)	/	/	/	BAO	10	Sigmoid	SS+CT	Died (23)
China Case 9	9	37	AP	II c	/	≥ 4	(+)	20%	(+)	(-)	(-)	(-)	(-)	(-)	/	(-)	(-)	(-)	(-)	198	BAO+(IA+DTIC)	NP	NP	NP	TF (15)
China Case 10	10	44	AP	III b	13	23	(-)	75%	(+)	/	(-)	/	/	(-)	/	/	(-)	/	/	/	OR	1	Pelvis	NP	Died (3)
China Case 11	11	52	AP	II b	12	14	/	< 5%	/	/	/	/	/	/	/	/	/	/	/	/	BAO+IAP	16	Pelvis	CT	Alive with tumor (34)
China Case 12	12	48	NP	I a	8	3~4	(-)	< 5%	(+)	/	(-)	(-)	/	(-)	/	/	(-)	/	/	/	BAO+IAP	NP	NP	NP	TF (45)
China Case 13	13	61	AP	III b	15	18	/	60%	(+)	/	(+)	(-)	(-)	(-)	/	/	(+)	/	/	/	OR	2	Pelvis	NP	Died (11)
Lee et al. [17]	14	69	VB	I	6	6~8	/	> 10%	/	/	/	/	/	/	/	/	(+)	/	/	/	BAO	/	/	/	/
Kruger et al. [18]	15	32	NP	I	7	8	/	5%-10%	/	/	/	(-)	(-)	(-)	/	(-)	/	/	/	116.1	OR	NP	NP	NP	TF (12)
Choi et al. [19]	16	44	AP	I a	18	17	/	< 1%	(+)	/	(-)	/	/	(-)	/	/	/	(-)	(-)	/	BAO+AP	NP	NP	NP	TF (120)
	17	34	AP	I b	13	8	/	20%	(+)	/	(-)	/	/	(-)	/	/	/	(+)	(+)	26.5	BAO+(VP16+IP)	NP	NP	NP	TF (60)
Testa et al. [20]	18	44	NP	III b	/	7	/		(+)	/	/	/	/	/	/	/	(+)	/	/	/	BAO+IA	NP	NP	NP	TF (50)
	19	50	NP	I a	/	5~7	/	12%	(+)	/	/	/	/	/	/	/	(+)	/	/	/	BAO	NP	NP	NP	TF (5)
Jimenez et al. [21]	20	55	VB	I c	23	< 1-2	/	60%	(+)	(-)	(-)	/	/	/	/	/	/	/	/	/	BAO+IA	14	Liver	SS	Alive with tumor (14)
Celyk et al. [5]	21	49	AP	III c	12	≥ 4	/	?	(+)	/	/	(+)	/	(+)	/	/	/	/	/	300	BAO+TP	36	Pelvis, liver	NP	Died (42)
McCluggage et al. [22]	22	61	AP	III c	7	12~15	/	/	(+)	/	(-)	(-)	/	(-)	/	/	(+)	/	/	196	BAO+CT	/	/	/	/
Prat et al. [2]	23	61	/	I a	10	4	/	/	/	/	/	/	/	/	/	/	/	/	/	/	BAO+RT	18	Liver	NP	Died (18)
	24	59	/	II b	/	8	/	/	/	/	/	/	/	/	/	/	/	/	/	/	BAO	1	Sigmoid	SS+RT	Died (4)
	25	42	/	II b	/	25	/	/	/	/	/	/	/	/	/	/	/	/	/	/	BAO	1	Ureter	RT	Alive with tumor (13)
	26	65	/	I a	/	10	/	/	/	/	/	/	/	/	/	/	/	/	/	/	OR	6	Pelvis	NP	Died (13)
	27	73	/	III	6	7	/	/	/	/	/	/	/	/	/	/	/	/	/	/	CT	2	Pelvis, Peritoneum	CT	Died (2)
	28	49	/	I a	/	5	/	/	/	/	/	/	/	/	/	/	/	/	/	/	BAO	44	Pelvis, Peritoneum	CT	Died (48)
Fukuda et al. [23]	29	54	AP	I a	22	3~6	/	5.4%-8.2%	(+)	/	(-)	(-)	(-)	(-)	/	(+)	(+)	/	/	/	OR	14	Pelvis	SS+AP	TF (22)
Iiboshi et al. [24]	30	38	NP	I a	5.2	20~25	/	/	(+)	/	(-)	/	(-)	(-)	/	/	/	/	/	/	OR	NP	NP	NP	TF (24)
Watanabe et al. [25]	31	44	NP	IV	12	< 4	/	/	(+)	/	(+)	(-)	(-)	(-)	(-)	/	/	/	/	/	OR+RT	/	/	/	Died (4)

**NP**, not performed; TF, tumor-free; **AP **(Symptom), abdominal pain; **VB**, vaginal bleeding; **OR**, oophorectomy; **BA0**, a total hysterectomy with bilateral adnexectomy and an omentectomy; **CT**, chemotherapy; **RT**, radiotherapy; **SS, second surgery; IA**, ifosfamide + adriamycin; **DTIC**, dacarbazine; **PVB**, Cisplatin + Vincristine + Bleomycin; **IAP**, ifosfamide + adriamycin + Cisplatin; **AP**, adriamycin + Cisplatin; **VP-16**, etoposide; TP, paclitaxel + cisplatin

### Pathologic Features

The tumors detected ranged from 5.2 - 23 cm for the greatest diameter (average, 11.5). The specimens from the Medical College of Shantou University (n = 3) and the Cancer Center, Sun Yat-sen University (n = 5) were solid tumors, and a cut of the surface of the tumor revealed tan-yellow discoloration and partial necrosis. Microscopically, spindle-shaped cells were densely arranged in a whirling pattern, with myxoid stroma and hemorrhages observed in some areas. In general, the tumor cells contained elongated hyperchromatic nuclei, and in some areas, round or oval nuclei were observed. In all 31 cases, the mitotic value ranged from 1 - 25 mitoses per 10 high powered fields (HPFs) examined. For example, 3/31 (10%) cases had a mitotic count < 4 per 10 HPFs, while for 18/31 (58%) cases the mitotic count was ≥ 4 and < 10 per 10 HPFs, and for 10/31 (32%) cases the mitotic count was ≥ 10 per 10 HPFs. For a subset of cases, the Ki-67 (MIB-1) proliferation index values were also available, and included values of < 10% for 5 cases, ≥ 10% and < 50% for 9 cases, and ≥ 50% up to 85% for 5 cases. Tumor cells from a subset of cases were also associated with strong expression of vimentin (22/22, 100%) and an absence of CD117 expression (0%, 5/5). Additional immunohistochemical assays that were performed for a subset of the cases examined included detection of smooth muscle actin (SMA) (2/18, 11%), desmin (1/13, 8%), epithelial membrane antigen (EMA) (0/11, 0%), S-100 (1/19, 5%), CD99 (0/6, 0%), CD34 (1/5, 20%), α-inhibin (7/15, 47%), estrogen receptor (ER) (1/6, 17%), and progesterone receptor (PR) (1/6, 17%) (Table 1).

### Univariate and multivariate analysis

Various clinicopathologic variables were also evaluated to identify potential prognostic factors for survival. Univariate analysis identified FIGO stage, Ki-67-positive cells, and therapy to be associated with the prognosis of patients with ovarian fibrosarcomas (*P *< 0.05). The 2-year OS and DFS rates for FIGO stage Ia-Ic, or FIGO stage IIa-IIIc, were 77.1% and 36.2% (*P *= 0.014), and 68.4% and 22.9% (*P *= 0.003), respectively in each case (Figure 1B). When the 2-year OS rate was evaluated in relation to the percentage of Ki-67-positive cells, the survival of patients with < 10% vs. ≥ 10% of Ki-67 positive cells was 100% and 27.2%, respectively (*P *= 0.042). Similarly, the 2-year DFS rate for the same categories of Ki-67 were 66.7% and 36.3%, respectively (*P *= 0.076) (Figure 1C). In terms of treatments received, the 2-year OS rate for patients receiving BAO, OR, or BAO + RT, was 27.9%, while patients receiving BAO + CT had a 2-year OS rate of 100% (*P *= 0.002). For the same categories of patient treatment, the 2-year DFS rates were 28.5% and 72.9%, respectively (*P *= 0.008) (Figure 1D). In contrast, patient age (2-year OS, *P *= 0.348; 2-year DFS *P *= 0.224), tumor size (2-year OS, *P *= 0.793; 2-year DFS, *P *= 0.666), and mitoses/10 HPFs (2-year OS, *P *= 0.426; 2-year DFS *P *= 0.830) were not found to be prognostic for survival (Table 2).

**Table 2:** The 2-year OS and DFS rates associated with ovarian fibrosarcoma patients and Cox proportional hazard regression analysis of patient survival based on clinical and pathologic factors

Variables	Case (%)	2 year's rate			2 year's rate			Cox (OS)				Cox (DFS)			
								
								Univariate		Multivariate		Univariate		Multivariate	
								
		OS	X2	P	DFS	X2	P	HR (95% CI)	P	HR (95% CI)	P	HR (95% CI)	P	HR (95% CI)	P
**Age (years)**															
< 45	15 (48.4)	66			60										
≥ 45	16 (51.6)	45.4	0.879	0.348	27.8	1.476	0.224	1.731 (0.540-5.545)	0.356			1.882 (0.658-5.382)	0.238		
**FIGO stage**															
I a - I c	14 (45.2)	77.1			68.4										
II a - III c	17 (54.8)	36.2	6.041	**0.014**	22.9	8.964	**0.003**	4.525 (1.210-16.916)	**0.025**	10.156 (2.021-51.023)	**0.005**	4.914 (1.538-15.704)	**0.007**	18.761 (1.674-210.288)	**0.017**
**Largest diameter (cm)**															
< 10	11 (45.8)	43.2			44.4										
≥ 10	13 (54.2)	54.7	0.069	0.793	38.9	0.187	0.666	0.844 (0.236-3.014)	0.794			1.302 (0.389-4.360)	0.669		
**Mitoses/10 HPFs**															
< 4	22 (71.0)	49.7			48										
≥ 4	9 (29.0)	70	0.632	0.426	41.7	0.046	0.83	0.545 (0.119-2.499)	0.435			1.132 (0.358-3.575)	0.833		
**Ki-67 (MIB-1)-positive**															
< 10	5 (26.3)	100			66.7										
≥ 10	14 (73.7)	27.2	4.148	**0.042**	36.3	3.138	0.076	51.792 (0.053-5058)	0.261			5.862 (0.668-51.416)	0.11		
**Therapy**															
BAO/OR/CT/BAO+RT/OR+RT	20 (64.5)	27.9			28.5										
BAO+CT	11 (35.5)	100	9.462	**0.002**	72.9	6.97	**0.008**	0.070 (0.008-0.574)	**0.013**	0.029 (0.003-0.319)	**0.004**	0.197 (0.052-0.741)	0.016	0.071 (0.005-0.992)	**0.049**

**HR**, hazard ratio; **CI**, confidence interval; **OS**, overall survival; DFS, disease-free survival; **CS**, cytoreductive surgery; **OR**, oophorectomy; **CT**, chemotherapy; **RT**, radiotherapy; **BA0**, a total hysterectomy with bilateral adnexectomy and an omentectomy

A role for multimodal therapy in patient prognosis was also examined. Due to the limited numbers of patients included in this study, the available cases were divided into two groups: those receiving BAO, OR, CT, OR + RT, or BAO+RT therapy, and those receiving BAO+CT. For these groups, 12/20 (60%) patients vs 1/11 (9.1%) patient died of their disease within 5 years, respectively. Furthermore, patients in the former group were associated with a poorer prognosis and a 2-year OS rate of 27.9%, compared to those in the latter group that were associated with a 2-year OS rate of 100% [HR, 0.07; 95% CI, 0.008-0.574; *P *= 0.013] (Figure 1D).

Cox proportional hazard regression analysis of patient survival based on clinical and pathologic factors was also performed. Multivariate analysis identified FIGO stage (Ia- Ic vs. IIa- IIIc, Hazard Ratio (HR) = 0.231, 95% CI of ratio = [0.072, 0.743], *P *= 0.007) and treatment (BAO/OR/CT/OR + RT/BAO+RT vs. BAO+CT, HR = 6.516, 95% CI of ratio = [1.974, 21.52], *P *= 0.008) to be significant independent prognostic factors for survival. In contrast, patient age, tumor size, mitosis events per 10 HPFs, and percentage of Ki-67-positive cells present were not found to be significant independent prognostic factors for survival (Table 2).

## Discussion

Ovarian fibrosarcoma is an extremely rare entity [1,2,11], and is considered to arise directly from stromal cells around the sex cord of ovarian follicles. However, the malignant transformation of a previous fibroma is also believed to be a potential origin of ovarian fibrosarcoma as well. These tumors can occur at any age, although they are mostly diagnosed in menopausal and postmenopausal women. Clinically, the presentation of this disease includes pelvic pain, abdominal enlargement, or awareness of an abdominal mass [1-3]. In addition, the majority of tumors exhibit areas of necrosis and haemorrhage, capsular disruption, as well as infiltrative margins that lead to adhesion of the tumor with other pelvic organs. For the 31 cases compared in this study, the median patient age was 49 years, and 19 patients presented at a hospital with abdominal pain, vaginal bleeding, or notable pelvic swelling.

Previous studies have reported a moderate to marked degree of pleomorphism to be associated with ovarian fibrosarcomas, with the number of mitotic figures observed ranging from 4-25 per 10 HPFs [1,2,12,13]. For example, in an evaluation of 17 cases of malignant, as well as cellular, fibromatous tumors of the ovary, Prat and Scully [2] classified these cases into two categories, those with 1-3 mitotic figures per 10 HPFs, which were designated as cellular fibromas, vs. those with greater than 4 mitoses per 10 HPFs, which were designated as fibrosarcomas. Usually, these criteria would not present a problem. However, the results of a recent study by Irving et al. indicate that mitotic activity is not a unique criteria for malignancy. For example, cases of ovarian fibromatous tumors were found to be associated with a very high mitotic count, whereas blunt nuclei, which usually do not exhibit an aggressive course of disease, were diagnosed as 'mitotically active cellular fibromas' instead of fibrosarcomas [14]. In our study, 18 cases were associated with a mitotic count ≥ 4 or < 10, and 10 cases had a mitotic count ≥ 10. Only three cases had a mitotic count < 4. However, in the latter cases, one patient developed a metastasis in the liver one year later, one patient died 4 months following the surgery, and one patient was tumor-free for 45 months.

In work by Tsuji et al. [3], the Ki-67 index values for fibrosarcomas were found to be higher than the Ki-67 values for fibromas. In doubtful cases, an assessment of the proliferative activity according to the MIB-1 labeling index was found to improve the accuracy of the diagnosis [3]. In the present study, there were three doubtful cases. However, when morphology, immunohistochemical results, and proliferative features were examined, a clear diagnosis of fibrosarcoma could be made. Furthermore, high levels of Ki67 (MIB-1) expression were consistent with a malignant diagnosis, despite a low visual mitotic rate that was also observed. However, when a multivariate analysis was performed, the presence of Ki-67-positive cells was not found to be a significant independent prognostic factor for survival.

The optimization of treatment strategies to improve patient outcome for patients diagnosed with an aggressive tumor such as ovarian fibrosarcoma, have not been identified. As a result, most patients experience a fatal outcome due to early metastasis via the bloodstream and tumor recurrences that usually occur within 2 years of diagnosis. Furthermore, there is no universally accepted treatment modality for ovarian fibrosarcoma as there is for epithelial ovarian cancers. For example, surgical resection for an ovarian fibrosarcoma can range from a simple adnexectomy, to a total hysterectomy with bilateral adnexectomy and an omentectomy. In many cases, post-surgical adjuvant chemotherapy or radiation is also required [15,16]. After reviewing several studies, Miles et al. reported that surgery did not prevent the recurrence of this disease regardless of the extent of surgery, and adjuvant chemotherapy and radiation therapy did not influence patient survival [16]. Moreover, there are few reports to indicate that adjuvant chemotherapy may improve patient survival rates, although Huang et al. reported that the use of MAID (mesna, doxorubicin, ifosfamide, and DTIC) for the treatment of ovarian fibrosarcoma has shown potential for prolonging patient survival [4]. Moreover, Celyk et al. reported that a regimen of paclitaxel plus cisplatin can improve the prognosis for patients with advanced stage tumors in some cases [11]. In the present study, multimodal therapy was evaluated for its capacity to improve patient prognosis, and BAO surgery followed by chemotherapy was associated with the greatest improvement in prognosis.

## Conclusion

In conclusion, high rates of Ki-67-positive cells, representing a high proliferation rate, may contribute to the malignant characteristic associated with ovarian fibrosarcomas. However, this is in contrast with the low visual mitotic rate associated with these tumors. The results of the present study indicate that FIGO stage and treatment modality may represent prognostic factors for patient survival, and BAO followed by adjuvant chemotherapy is associated with an improved treatment outcome. In addition, although this study was retrospective in design, and included a limited number of patients, it is one of the largest series reported to date. As such, these results provide valuable insight into a challenging and rare disease, and contribute to the limited body of knowledge that is currently available for this aggressive tumor type.

## Competing interests

The authors declare that they have no competing interests.

## Authors' contributions

**L H **participated in the study design, data collection and analysis, and participated in drafting and revising the manuscript. **LM L **participated in the study design, data collection and analysis, and participated in drafting and revising the manuscript. **HY W **participated in the study design, and critical revision of the manuscript. **M Z **conceived the study, was responsible for its design and coordination, participated in the analysis and interpretation of the data, as well as in drafting and revising all versions of the manuscript. All authors read and approved the final manuscript.

## Pre-publication history

The pre-publication history for this paper can be accessed here:

http://www.biomedcentral.com/1471-2407/10/585/prepub
